# A Paediatric Case of Sphenoid Sinusitis and Resultant Secondary Masticator Space Abscess Requiring Surgical Management

**DOI:** 10.7759/cureus.94843

**Published:** 2025-10-18

**Authors:** Nazia Hossain, Chi H Song, Maya Stewart-Rizza, Emma Wates, James Sloane

**Affiliations:** 1 Oral and Maxillofacial Surgery, Kent Surrey Sussex Postgraduate Dental Deanery, Surrey, GBR; 2 Oral and Maxillofacial Surgery, Royal Surrey NHS foundation Trust, Guildford, GBR; 3 Ear, Nose, and Throat (ENT), Royal Surrey NHS foundation Trust, Guildford, GBR; 4 Oral and Maxillofacial Surgery, Kent Surrey Sussex Postgraduate Deanery, Surrey, GBR

**Keywords:** deep space infection, endoscopic sphenoidotomy, masticator abscess, multi-disciplinary teams, paediatric facial pain, sphenoid sinusitis, streptococcus intermedius

## Abstract

Paediatric sphenoid sinusitis is rare and potentially life-threatening, with delayed diagnosis increasing the risk of intracranial complications. Masticator space abscesses are also uncommon, often arising from odontogenic infection. We describe the case of a healthy early adolescent male patient with sphenoid sinusitis complicated by a secondary masticator space abscess without odontogenic involvement, with early erosion of the lateral pterygoid plate posited as a possible route of infection spread. The patient presented to the emergency department with nonspecific right-sided facial pain, swelling, and transient facial weakness. Urgent magnetic resonance imaging (MRI) with contrast confirmed sphenoid sinusitis.  He had an initial joint admission under both Paediatric and Ear, Nose, and Throat (ENT) surgical teams. In the month following initial discharge, he re-presented twice with subsequent imaging revealing a right masticator space abscess, and thereafter, recurrence with early erosion of the right lateral pterygoid plate. This required sphenoidotomy and abscess drainage on both occasions, necessitating involvement of the Oral and Maxillofacial Surgery (OMFS) team. With extended targeted intravenous antibiotic therapy, a full recovery was made. There are no cases of sphenoid sinusitis leading to a secondary masticator space abscess without odontogenic involvement described in the available literature. The anatomical separation and typical routes of infection spread make this complication exceedingly rare. This case illustrates the importance of high clinical suspicion, advanced imaging, and prompt, coordinated multidisciplinary management.

## Introduction

The underlying cause of facial pain can be difficult to accurately diagnose in children due to the broad range of possible differential diagnoses [1]. The deep location of the sphenoid sinus and often vague presenting symptoms can lead to a delay in definitive diagnosis and, therefore, timely, accurate management. Typical symptoms of sphenoid sinusitis are often nonspecific and include facial pain and headaches as well as fever, nasal discharge, and/or neurological signs in complex disease [2]. Confirming a diagnosis of sphenoid sinusitis relies on a wide range of investigations, including nasal endoscopy, CT of paranasal sinuses, and MRI to exclude other intracranial pathology. The basis of management typically consists of nasal decongestants, intranasal corticosteroids, and intravenous (IV) or oral antibiotics. Surgical drainage via endoscopic transnasal sphenoidotomy may also be needed in refractory cases [3].  

Due to the sphenoid sinuses’ proximity to vital neurovascular structures, early and successful management is key in preventing further complications such as blindness, ophthalmoplegia, cavernous sinus thrombosis, meningitis, and possible spread to deep neck spaces [4,5]. The masticator space is anatomically separated from the sphenoid sinus by numerous bony and fascial barriers, making direct lateral extension exceedingly unlikely. Existing literature almost exclusively demonstrates that masticator space abscesses arise from odontogenic sources, with possible direct extension through contiguous fascial planes or bone destruction [6]. Here, we describe the case of a healthy early adolescent male with sphenoid sinusitis complicated by a secondary masticator space abscess without odontogenic involvement. The absence of any previously reported case of infection spread from the sphenoid sinus to the masticator space highlights the extreme rarity of the case we present. 

## Case presentation

A previously fit and healthy early adolescent male patient presented to the emergency department of our district general hospital with a three-day history of worsening, nonspecific right-sided facial pain. Two days prior, the patient reported transient right ptosis and photophobia, which had resolved on clinical review. He had attended a dental hygienist appointment seven days prior.  

The facial pain was described as constant, with further intermittent episodes of severe, sharp, and shooting pain extending from the right temporomandibular joint radiating down to the jaw. The pain worsened with movement and was alleviated with rest and lying flat. The patient had experienced one episode of vomiting. Taste, vision, and hearing were reported as normal. There was no history of recent trauma, fevers, neck stiffness, or rashes. No voice changes were noted. 

Past medical history consisted of occasional bilateral migraines without aura from the age of seven, which were managed with simple analgesia on his general practitioner’s advice. He had an umbilical hernia repair and circumcision operation at age two and took chlorphenamine as needed during hay fever season. He had a penicillin allergy in the form of a rash and wheezing, and he weighs 49.7 kg. The patient lived at home with his parents and dog. He regularly visited his general dental practitioner and has had no treatment to date apart from routine dental hygiene visits for superficial cleaning. In terms of his birth history, he was born at 37 weeks and five days and required phototherapy for jaundice and vitamin D5. He has had all recommended vaccinations and has met all his developmental milestones.  

On initial examination, the patient had a Glasgow Coma Scale (GCS) score of 15. Cranial nerve, ear, nose, and throat, intra-oral, and neck examination were unremarkable. There was no facial swelling or erythema; however, right-sided facial hyperaesthesia was present. There was no intra-oral swelling, pus, or gingival erythema. There were no clinical concerns on examination of the oropharynx and neck. Initial vital observations recorded in the emergency department concluded a Paediatric Early Warning Score (PEWS) of 0. This included a temperature of 37.2 °C, heart rate of 99 beats/minute, blood pressure of 115/78 mmHg, respiratory rate of 24 breaths/minute, and oxygen saturation of 100% on room air. The patient was admitted from the emergency department under Paediatrics for further management and work-up, given the symptom severity. 

Investigations

**Figure 1:**
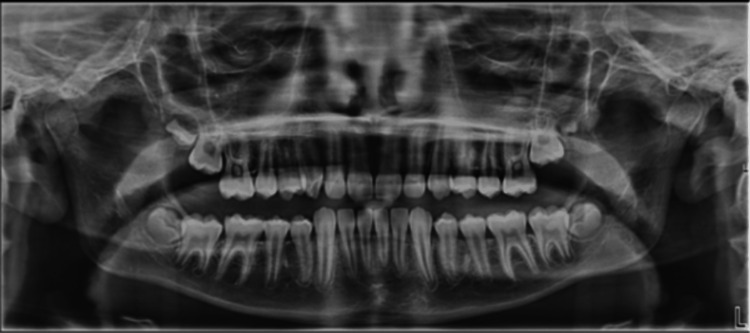
Initial orthopantomogram confirming no dental pathology

Routine blood tests, including full blood count, urea and electrolytes, and C-reactive protein (CRP) were taken. They showed a haemoglobin level of 117 g/L (130-180 g/L), CRP of 123 mg/L (0-5 mg/L), a white cell count of 9.5×10^9^/L (4-11x10^9^/L) and neutrophils of 6.1×10^9^/L (2-7.5x10^9^/L). Urea and electrolytes were within normal limits. Plain film imaging in the form of an orthopantomogram (OPG) (Figure 1) was taken, which confirmed no dental pathology. 

**Figure 2:**
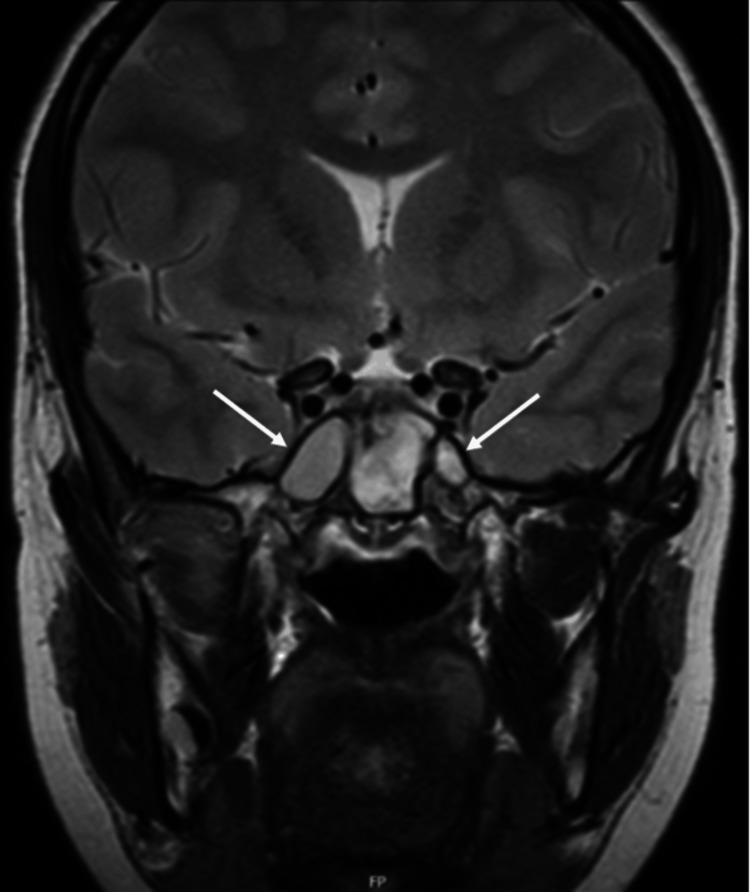
Initial coronal slice non-contrast MRI head showing extensive inflammatory changes within the sphenoid sinuses bilaterally, worse on the right

An MRI of the head (Figure 2) was taken on day two of the initial admission, which was reported by a consultant head and neck radiologist, showing no trigeminal nerve abnormalities. Extensive inflammatory changes were seen within the sphenoid sinuses bilaterally. The maxillary sinuses were clear aside from minor mucosal thickening. 

Differential diagnosis 

An initial working diagnosis of trigeminal neuralgia [7] due to the unilateral nature of the facial pain following maxillary distribution was made by the admitting Paediatrics team prior to imaging, postulating a possible unknown secondary infection. However, the definitive diagnosis of sphenoid sinusitis was made on receiving the initial MRI report and prompted local ENT input for further management. Other possible differential diagnoses included temporomandibular joint (TMJ) septic or reactive arthritis due to the tender right TMJ swelling, but the MRI findings did not support this. A dental abscess was also considered due to the unilateral nature of the facial pain, together with a history of a recent dental hygiene appointment, but the OPG findings did not support this diagnosis.  

Treatment

Initial management included hospital admission for IV ceftriaxone (4 g once a day in the afternoon) and IV aciclovir as cover for possible infections. Following the MRI showing the working diagnosis of sphenoid sinusitis, aciclovir was discontinued. Betamethasone nasal drops (0.1%, two drops twice a day) and Otrivine nasal spray (0.1%, one spray three times a day) were prescribed for topical use, and the IV ceftriaxone was continued. The patient was discharged following clinical improvement alone after a five-day hospital admission with betamethasone nasal drops (0.1%, two drops twice a day for 14-21 days) and Otrivine nasal spray (0.1%, one spray three times a day for seven days). 

**Figure 3:**
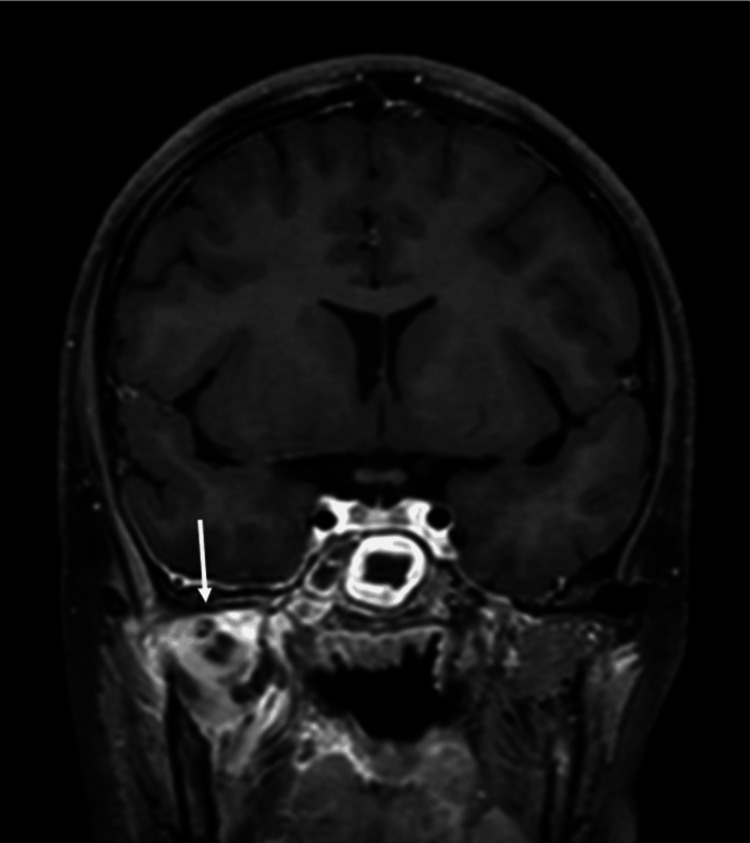
Second hospital admission. Coronal slice MRI head with contrast showing a 2.4 x 2.2 x 3.3 cm multiloculated abscess in the right masticator space

The patient attended the maxillofacial clinic for a planned review nine days following discharge from the hospital due to the atypical jaw pain on initial admission not thought to be related to sphenoid sinusitis. He was admitted from the clinic due to worsening symptoms in the form of vomiting and uncontrolled right-sided facial pain. This was a joint admission under Paediatrics, Oral and Maxillofacial Surgery (OMFS), and ENT. On examination, the patient described pain over the right temporomandibular joint (TMJ) and trismus with a maximum mouth opening of 20 mm. Blood test results included a CRP of 82 mg/L (0-5 mg/L), white cell count of 15.7 ×10^9^/L (4-11x10^9^/L), and neutrophil count of 12.5×10^9^/L (2-7.5 x10^9^/L). A repeat MRI head with contrast (Figure 3) was carried out the following day. This showed a 2.4 x 2.2 x 3.3 cm multiloculated abscess in the right masticator space, deemed to have tracked down from the sphenoid sinus, inflammation of the right TMJ synovium, and mild dural enhancement of the right temporal lobe suggestive of reactive inflammation. 

The MRI findings of dural inflammation were discussed with our local specialist neurosurgery unit, who confirmed that no neurosurgery input was needed. Arrangements were made for the patient to go to the theatre for urgent drainage under general anaesthetic as a joint case with OMFS and ENT. ENT carried out endoscopic bilateral sphenoidotomy via a nasal approach. Incision and drainage of the right masticator abscess was carried out via an intra-oral approach. A mucoperiosteal flap was raised around the last standing molar with a distal relieving incision. Buccal and lingual flaps were raised. The masticator space was accessed deep to the lingual flap. Pus was found in this space, and a sample was taken for microbiology, culture, and sensitivity testing. A corrugated drain was placed and secured intra-orally. Medical management during this second admission included IV ceftriaxone (4 g once a day in the afternoon) and oral metronidazole (400 mg three times a day). Nasal douching (syringe of saline 0.9% 10 ml twice a day before steroid drops), betamethasone nasal drops (0.1%, two drops twice a day), xylometazoline hydrochloride nasal spray (0.1%, one spray three times a day), and chlorhexidine mouthwash (0.2%, 10 ml, twice a day) were also given. 

The corrugated intra-oral drain was removed after four days, and the patient was discharged five days postoperatively with a five-day course of oral clarithromycin (500 mg twice a day) and oral metronidazole (400 mg three times a day). Other discharge medications included chlorhexidine mouthwash (0.2%, 10 ml twice a day for two days), betamethasone nasal drops (0.1%, two drops twice a day for five days), and dihydrocodeine (30 mg, up to six times a day as needed for three days). A follow-up review with ENT was booked for three weeks. The culture and sensitivity testing results showed light growth of *Streptococcus intermedius* that was sensitive to penicillin but resistant to clindamycin after seven days of incubation. It is to be noted that these results were not available at the time of discussion regarding appropriate discharge antibiotics with local microbiology on day five post-operation. 

**Figure 4:**
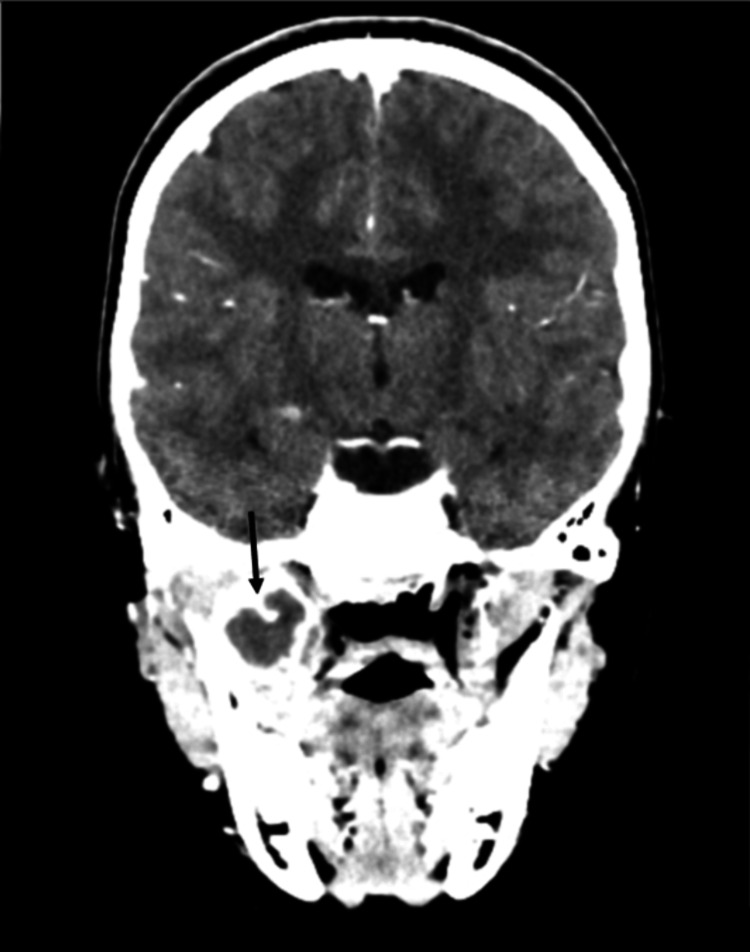
Third hospital admission. Coronal slice CT head with contrast showing a recurrent abscess within the right masticator space

**Figure 5:**
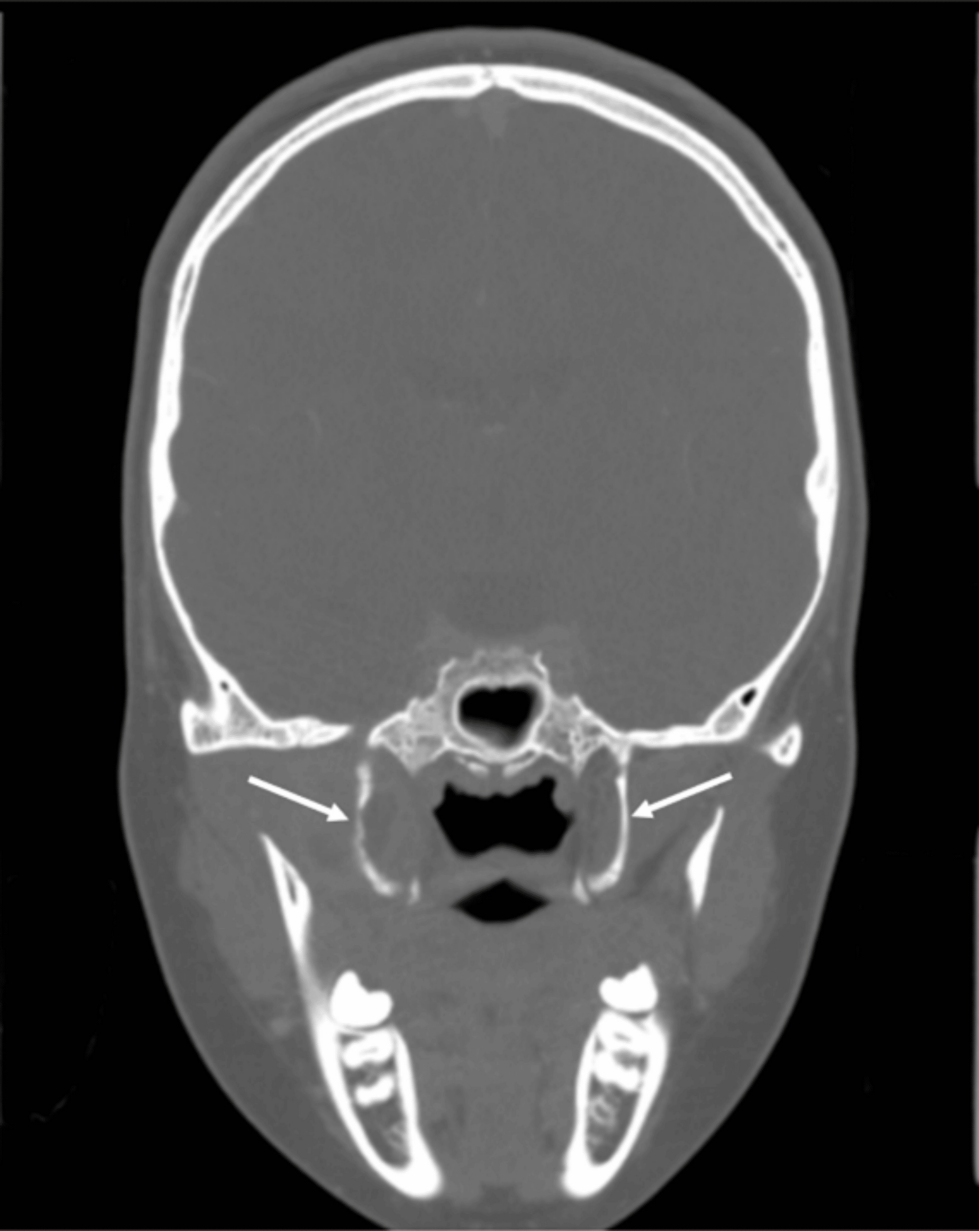
Third hospital admission. Coronal slice CT head with contrast showing early erosion of the lateral pterygoid plates, worse on the right

The patient re-presented on his own accord to the emergency department 17 days following discharge with ongoing right-sided facial pain, swelling, trismus, and fevers. A computed tomography (CT) head scan with contrast was taken. This showed a recurrent abscess within the right masticator space (Figure 4) and some early erosion of the right lateral pterygoid plate (Figure 5), with tracking fluid into the pterygopalatine fossa and right TMJ. 

The patient was returned to emergency theatres for repeat urgent incision and drainage of the right masticator abscess and re-exploration of the right sphenoid sinus. The right sphenoid sinus was accessed transnasally, and the neo-ostium was widened. No pus was seen, but the mucosa was inflamed within the sinus. A thorough washout was done. Incision and drainage were carried out of the right masticator abscess via an intra-oral approach. This time, a maxillary sulcal incision and an inferior incision along the external oblique ridge were made to raise a mucoperiosteal flap. This allowed for exploration of the infratemporal fossa and the breakdown of loculations of pus around the pterygoid plates. A further pus swab was taken for microbiology, culture, and sensitivity testing. Thorough debridement was performed using povidone-iodine and saline. Two corrugated drains were placed in the infratemporal fossa and medial to the pterygoid plates, which were secured with silk sutures intra-orally.

**Figure 6:**
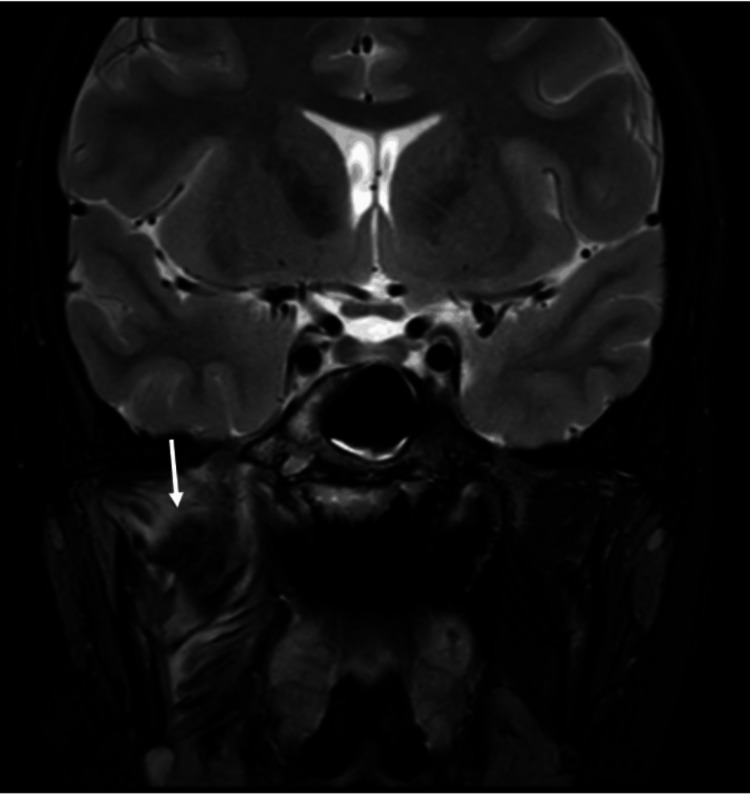
Third hospital admission. Coronal slice repeat MRI head and sinus with contrast showing significant inflammatory changes in the right masticator space but no identifiable collection and improvement of localised inflammatory changes of the dura and right TMJ

Medical management included IV ceftriaxone (4 g once a day in the afternoon), oral metronidazole (400 mg three times a day), and betamethasone nasal drops (0.1%, two drops twice a day) as an inpatient while awaiting culture results. The microbiology culture grown from re-drainage of the right masticator space abscess demonstrated the same light growth of *S. intermedius* sensitive to penicillin with resistance to clindamycin. A repeat MRI head and sinus with contrast was taken prior to discharge (Figure 6). This showed that there were significant inflammatory changes in the right masticator space but no identifiable collection. It was noted that the localised inflammatory changes of the dura and right TMJ had also improved. 

The patient was discharged 14 days after the third hospital admission with betamethasone nasal drops (0.1%, two drops twice a day for seven days), oral metronidazole liquid (400 mg three times a day for 14 days), and IV ceftriaxone (4 g once a day in the afternoon for 14 days). The patient was advised to carry out some gentle jaw exercises using tongue spatulas to help improve mouth opening. 

Outcome and follow-up

Although this patient required both repeat surgical and medical management of the resultant masticator abscess, having failed to respond to the initial medical management of the sphenoid sinusitis alone, the patient had a good outcome as the symptoms resolved completely. The blood tests that were last taken 14 days after the third hospital admission showed a CRP <1 mg/L (0-5 mg/L) and white cell count of 32×10^9^/L (4-11x10^9^/L). 

The hospital liaised with the paediatric community services, which enabled outpatient parenteral antimicrobial therapy (OPAT) in the form of once daily IV ceftriaxone to be administered in the community setting. This required a midline catheter to be inserted under general anaesthesia due to an unsuccessful attempt under sedation. 

Outpatient review with ENT was arranged two weeks following discharge to monitor repeat blood tests and discuss the recommended antibiotic duration. Repeat blood tests showed a haemoglobin level of 152 g/L (130-180 g/L), CRP of <1 mg/L (0-5 mg/L), a white cell count of 5.2×10^9^/L (4-11×10^9^/L) and neutrophils of 2.3×10^9^/L (2-7.5x10^9^/L). Urea and electrolytes were within normal limits. The full six weeks of IV ceftriaxone were agreed to be completed following discussion with Paediatric Infectious Diseases at a tertiary centre and local microbiology. A follow-up MRI head with contrast undertaken two and a half weeks after the third hospital discharge revealed a completely clear right sphenoid sinus with no evidence of intracranial complication. It was noted that there was some ongoing inflammation involving the right masticator space, but with no drainable collection. 

Table 1 highlights the key events. 

**Table 1:** Summary of clinical course, imaging progression, and management OMFS: Oral and Maxillofacial Surgery; CRP: C-reactive protein; OPG: orthopantomogram; WCC; white cell count; TMJ: temporomandibular joint

Timeline	Symptoms & Clinical Findings	Imaging Findings	Management / Interventions
Day 1 (Initial Presentation)	3-day history of worsening right facial pain, transient ptosis, photophobia (resolved), and facial hyperaesthesia. No swelling or fever. CRP of 123 mg/L	MRI head (non-contrast): Bilateral sphenoid sinus inflammatory changes, worse on the right. No dental pathology on OPG.	Admitted under Paediatrics. IV ceftriaxone, aciclovir (discontinued after MRI), betamethasone nasal drops, and Otrivine nasal spray. Discharged after 5 days on topical therapy.
Day 14 – Day 19 (Planned review)	Worsening right-sided facial pain, trismus (20 mm), vomiting. CRP of 82 mg/L and WCC of 15.7×10⁹/L.	MRI head (contrast): 2.4 × 2.2 × 3.3 cm multiloculated right masticator space abscess; dural enhancement; right TMJ synovial inflammation.	Readmitted under joint Paediatrics/ENT/OMFS. Endoscopic bilateral sphenoidotomy and intra-oral drainage of masticator abscess. One intra-oral corrugated drain placed. IV ceftriaxone, oral metronidazole, nasal douching, and topical steroids. Discharged on day 5 post-op with clarithromycin, metronidazole, chlorhexidine mouthwash, topical steroids, and analgesia.
Day 31 (Re-presentation)	Recurrence of right facial pain, swelling, trismus, and fever.	CT head (contrast): Recurrent right masticator abscess with early erosion of right lateral pterygoid plate, tracking fluid to TMJ and pterygopalatine fossa.	Repeat surgery: Re-exploration and drainage of masticator abscess and right sphenoid sinus washout. Two intra-oral corrugated drains placed. Continued IV ceftriaxone, oral metronidazole, and topical steroids.
Day 41 - 45 (Pre-discharge imaging)	Clinical improvement.	MRI head (contrast): No drainable collection; improving dural and TMJ inflammation.	Discharged on 14 days IV ceftriaxone (via OPAT), oral metronidazole, and betamethasone drops. Advised gentle jaw exercises.
2-week Follow-up	Asymptomatic; normal bloods (CRP <1 mg/L, WCC 5.2×10⁹/L).	MRI head (contrast): Clear sphenoid sinus, no intracranial complications, mild residual soft-tissue inflammation.	Agreed continuation to 6-week IV ceftriaxone

## Discussion

The clinical progression observed in this case (an early adolescent male child developing sphenoid sinusitis and a secondary masticator abscess without odontogenic involvement) represents a novel and previously undocumented pathway of infectious spread in both paediatric and adult populations. 

In the paediatric population, isolated sphenoid sinusitis is uncommon due to the relatively late development of sphenoid sinuses and poor pneumatisation in early childhood. Radiological studies show that around 90% of children have signs of sphenoid pneumatisation by age four and 100% by the age of 10 [8]. Moreover, the relatively lower number of mucous glands found in the sphenoid sinus may contribute to reducing susceptibility to infections [9]. One review estimated that isolated sphenoid sinus disease only accounts for around 0.4% of paediatric sinusitis and up to 1-2.7% in the overall adult and paediatric general population with paranasal sinus pathology [9]. 

Facial swelling, transient facial weakness, and unilateral trigeminal distribution of pain are not typical of sphenoid sinusitis presentation. Sphenoid sinusitis in children usually presents insidiously. Typical symptoms include headache, often retro-orbital, frontal, or generalised (99% of cases), fever (50% of cases), and ocular symptoms (19.7% of cases) [10]. Sinonasal symptoms (congestion, rhinorrhoea) are less common than with other subtypes of sinusitis. The patient in this case did not at any point experience headaches or congestion and had been apyretic on initial presentation. Documented case reports on acute isolated sphenoid sinusitis highlight frequent difficulties in obtaining accurate and timely diagnosis due to vague and variable presenting symptoms as well as significant diversity in clinical courses [11]. 

Neurological signs such as cranial nerve palsies or decreased level of consciousness occur in around 13-28% of patients and usually indicate complex spread of infection [10]. Severe cases may result in cranial neuropathies or intracranial complications, most often resulting from bony erosion or superior extension rather than lateral spread to the masticator space [11]. In practice, a persistent or severe headache in children with visual changes or altered mental status should prompt consideration of sphenoid sinusitis. 

The often atypical nature of sphenoid sinusitis, supported by the rarity of this case, helps to emphasise the usefulness of early imaging not only in surgical planning but diagnosis. CT of the sinuses should be obtained whenever sphenoid sinusitis is suspected, which will demonstrate sinus opacification and any bony erosion. In Elden et al.’s series, CT detected disease in every case, with MRI demonstrating a complementary role due to superior soft tissue contrast and ability to better map the extent of infection beyond the sinus [12]. Accordingly, current practice is to employ both modalities when sphenoid sinusitis is confirmed. CT is preferred for defining bony anatomy and sinus drainage pathways, whereas MRI should be reserved for suspected complications [12].

*S. intermedius* (part of* *the*Streptococcus anginosus* group (SAG)) has been increasingly recognised in children with complicated sinusitis. Though part of normal oral flora, when pathogenic, *S. intermedius* is frequently associated with deep-seated abscesses [13]. Its isolation in this context supports a pathogenic role in the aggressive clinical course, including rapid extension and osteolysis. Fortunately, standard beta-lactam antibiotics are highly effective (92.6% sensitive), and one paediatric series showed all *S. intermedius* isolates were sensitive to ceftriaxone, levofloxacin, chloramphenicol, vancomycin, and linezolid. In contrast, it showed high resistance to erythromycin (73.3%) and clindamycin (76.7%) [14]. The presence of *S. intermedius* should prompt consideration of prolonged intravenous antibiotic therapy and close monitoring for further complications. 

The interpretation of culture results in this case is limited by prior antibiotic exposure. A minimum of seven days of intravenous ceftriaxone before sampling may have reduced culture yield, potentially resulting in incomplete microbiological data. The isolation of *S. intermedius*, while significant, does not exclude polymicrobial infection; therefore, empirical therapy was continued to cover typical pathogens, including anaerobes and *Staphylococcus* species.

Due to the severity of the infection reported and repeated return to theatre for drainage, six weeks of intravenous antibiotics were administered as per both local microbiology and tertiary Paediatric Infectious Diseases advice. Whilst prolonged courses of IV antibiotic therapy are not routinely given for uncomplicated sinusitis, it is recommended in cases with intracranial spread, deep space abscesses, or, in this case, recurrent infection. A retrospective study of complicated sinusitis showed that a prolonged course of antibiotics (usually four to six weeks) correlated with reduced recurrence and need for surgical intervention [15].  

Although there is no standard guideline for surgical treatment in sphenoid sinusitis, the consensus is that early endoscopic sphenoidotomy is indicated if complicated with neurological or visual compromise. Otherwise, surgical drainage is reserved for patients who are not responding to maximal medical therapy [12]. One paediatric case series showed that around one-third of children ultimately required surgical drainage [11]. Optimal management of sphenoid sinusitis balances medical treatment with timely surgery. In general, children are started on broad-spectrum intravenous antibiotics and supportive care. If a child fails to improve quickly or if any red-flag signs appear, endoscopic sphenoidotomy is indicated [11,12]. 

Sphenoidotomy is proven to be effective, and the need to return to theatre for repeat sphenoidotomy is rare in paediatric cases. As shown in a review, sphenoid sinusitis was resolved following the first sphenoidotomy without the need for re-operation [16]. Persistent or recurrent symptoms are often related to inadequate initial drainage, unusual anatomy, or unresolved complications such as deep space abscesses. Re-operation should warrant a review of investigation results, such as anatomical variation or access and culture results, to exclude missed pathology or ongoing areas of infection. 

The masticator space is considered anatomically separated from the sphenoid sinus. The sphenoid sinus is located deep within the sphenoid bone, with its lateral wall formed by thick bone, including the greater wing and the pterygoid processes, which act as robust barriers to lateral spread of infection. The masticator space is a deep facial compartment bounded medially by the lateral pterygoid plate and laterally by the ramus of the mandible, thus separated from the sphenoid sinus by these significant bony structures [17]. Infection from the sphenoid sinus typically spreads to adjacent structures, rather than laterally into the masticator space. In this case, the most plausible anatomical route of spread is direct extension through the lateral wall of the right sphenoid sinus with subsequent bony erosion of the right lateral pterygoid plate, which was seen on the CT taken during the patient's third and final admission but not on the earlier MRIs. In paediatric patients, MRI is often preferred to minimise radiation exposure. This discrepancy likely highlights the differences in sensitivity between the imaging modalities, as CT is superior in detecting fine bony changes while MRI excels at soft tissue and marrow pathology [18]. The possibility of haematogenous dissemination is not recognised in the available medical literature as a mechanism for the development of a masticator space abscess linked to sphenoid sinusitis. The occurrence of early pterygoid plate erosion as a conduit for infection is highly unusual and not previously described in paediatric or adult case series, highlighting the unique nature of this case and the need for high clinical suspicion and advanced imaging in atypical presentations.

If left untreated or under-treated, sphenoid sinusitis can lead to serious vascular and intracranial complications. Given its proximity to critical structures such as the cavernous sinus, internal carotid artery, and base of the skull, complications could arise rapidly. One series of severe cases showed that up to 33% of patients developed subdural empyema and 27% developed intracranial abscesses; complete recovery occurred in 71% of patients, and there was a 6% chance of fatality [19]. Therefore, early recognition and intervention remain crucial. 

## Conclusions

As far as we are aware, this is the first documented case of sphenoid sinusitis complicated by a secondary masticator space abscess without odontogenic involvement in either the adult or paediatric populations, with early erosion of the lateral pterygoid plate as a possible route of spread, as shown on later CT imaging. Repeat MRI showed the isolated sphenoid sinusitis had spread to the masticator space, resulting in an abscess requiring repeat open surgical drainage. The isolation of *S. intermedius*, a member of the SAG, underscores the aggressive nature of this infection and its established association with deep space abscesses and bony destruction in children. 

This case highlights the importance of early diagnosis, advanced imaging, and a prompt, coordinated multidisciplinary management: OMFS and ENT team for adequate surgical drainage (open incision and drainage of the secondary masticator space abscess and endoscopic sphenoidotomy), neurosurgical input in management of dural inflammation as well as microbiology and Paediatric Infectious Diseases guidance in ensuring adequate duration of antimicrobial therapy. The delayed recognition of bony erosion, possibly limited by imaging modality choice, and the need for repeated intervention emphasises that atypical presentations may indicate aggressive disease and therefore require prompt, coordinated care to optimise outcomes and ensure full recovery is made as seen here. This report expands the understanding of possible infectious pathways and complications in sphenoid sinusitis, illustrating effective strategies to optimise outcomes. 
